# Facile Synthesis of Nitrogen and Oxygen Co-Doped Clews of Carbon Nanobelts for Supercapacitors with Excellent Rate Performance

**DOI:** 10.3390/ma11040556

**Published:** 2018-04-04

**Authors:** Liang Yu, Shaozhong Zeng, Xierong Zeng, Xiaohua Li, Hongliang Wu, Yuechao Yao, Wenxuan Tu, Jizhao Zou

**Affiliations:** 1 Shenzhen Key Laboratory of Special Functional Materials & Shenzhen Engineering Laboratory for Advance Technology of Ceramics, College of Materials Science and Engineering, Shenzhen University, Shenzhen 518060, China; 2150120414@email.szu.edu.cn (L.Y.); yizhumeng@126.com (S.Z.); zengxier@szu.edu.cn (X.Z.); lxh@szu.edu.cn (X.L.); whl@szu.edu.cn (H.W.); 2150120428@email.szu.edu.cn (Y.Y.); jetyulianjie@163.com (W.T.); 2 JANUS (Dongguan) Precision Components Co., Ltd., Dongguan 523841, China

**Keywords:** co-doped, carbon nanobelts, hierarchical, supercapacitors

## Abstract

Facile synthesis of carbon materials with high heteroatom content, large specific surface area (SSA) and hierarchical porous structure is critical for energy storage applications. In this study, nitrogen and oxygen co-doped clews of carbon nanobelts (NCNBs) with hierarchical porous structures are successfully prepared by a carbonization and subsequent activation by using ladder polymer of hydroquinone and formaldehyde (LPHF) as the precursor and ammonia as the activating agent. The hierarchical porous structures and ultra-high SSA (up to 2994 m^2^ g^−1^) can effectively facilitate the exchange and transportation of electrons and ions. Moreover, suitable heteroatom content is believed to modify the wettability of the carbon material. The as-prepared activated NCNBs-60 (the NCNBs activated by ammonia at 950 °C for 60 min) possess a high capacitance of 282 F g^−1^ at the current density of 0.25 A g^−1^, NCNBs-45 (the NCNBs are activated by ammonia at 950 °C for 45 min) and show an excellent capacity retention of 50.2% when the current density increase from 0.25 to 150 A g^−1^. Moreover, the NCNBs-45 electrode exhibits superior electrochemical stability with 96.2% capacity retention after 10,000 cycles at 5.0 A g^−1^**.** The newly prepared NCNBs thus show great potential in the field of energy storage.

## 1. Introduction

In recent years, people continue to face enormous energy challenge. Exploring sustainable energy and efficient energy storage devices is a pressing issue for researchers [1]. The electrical double layer supercapacitor (EDLC) is a new energy storage device that is playing an increasingly important role due to its long cycling stability, high energy density, and fast charge/discharge rate [2,3].

Electrode materials affect the performance of supercapacitors [4,5]. Although some transition metal oxides (RuO_2_, MnO_2_ and NiO and so on) and some conducting polymers are used for electrode materials [6,7,8]. Porous carbon materials are the most studied for supercapacitors owing to their low cost, stability, environmental friendliness, and easy availability [9]. Among various types of carbon materials, activated carbon has been considered the most ideal material for supercapacitor electrodes. nevertheless, the pore size distribution of most activated carbon materials is not appropriate and the utilization of the specific surface area (SSA) is quite low, which limits its performance and, in particular, its power density [10,11]. Carbon nanotubes (CNTs) have a vast, ion-accessible surface area and perfect electronic conductivity, which can provide outstanding rate performance [12]. Nevertheless, the specific capacitance of pure CNTs is only 20–80 F g^−1^ [13,14], which is mainly due to the small SSA of CNTs. Graphene has good electrical conductivity and a high theoretical SSA, and has been regarded as being highly competitive in the field of supercapacitors [12]. However, the strong Vander Waals force tends to restack the graphene sheets, leading to depressed performance compared with theoretical value [15,16]. Although many methods have been used to enhance the performance of graphene supercapacitor electrodes, the effect has not been very satisfactory. Therefore, it is an urgent task to explore new porous carbon materials for supercapacitors [17].

Although porous carbon material is the ideal candidate for supercapacitor electrodes, porous carbon still has many disadvantages [18,19]. Especially its electrical conductivity, which decreases with increasing porosity [20]. The energy density of the supercapacitor is calculated by the following formula [21]: $E=CV^{2}/2\;.$ Therefore, increasing the capacitance and/or working voltage of the carbon material electrode can improve the energy density of the supercapacitor. Generally, if the carbon material has a large SSA, reasonable pore size distribution, excellent conductivity and wettability, then the supercapacitor with such carbon electrodes performs well [22]. In the past several decades, various methods have been used to modify the performance of porous carbon materials for supercapacitors, most of which are improving SSA [23,24]. Carbon materials with a large SSA can provide a more active interface for the adsorption of ions, which can increase the capacity of the supercapacitors. KOH activation is considered to be an effective method. The carbon activated by KOH has a SSA of more than 3000 m^2^ g^−1^ [25]. However, the ultra-large SSA of the activated carbon material is essentially contributed by the micropores and due to the intrinsic hydrophobic properties of carbon-based materials, the internal surface is poorly infiltrated by electrolytes and the SSA utilization is limited. Therefore it is difficult to achieve an expected specific capacity [26]. Heteroatom doping is recognized as an effective way to improve surface physics and chemical activity, which can improve the wettability and conductivity of porous carbon materials [27]. The effect of nitrogen doping is particularly remarkable and the electronegativity difference between N and C atoms provides a larger polarized surface, which increases the wettability of the carbon material surface and ensues a faster transmission rate of electrolyte ions in the mesopores and micropores. This improves the effective utilization of the surface area. In addition, oxygen doping has a similar effect. Therefore, nitrogen and oxygen co-doped porous carbon with a high SSA can significantly improve the performance of the supercapacitor [28]. 

The hierarchical pore structure of carbon is considered to be the key to achieving excellent rate performance for supercapacitors [29]. The capacitance value of the carbon material to a great extent depends on the SSA, pore size distribution, and the connectivity between the pores [30]. In the hierarchical pore system, the micropores provide a large SSA, while the macropores can serve as electrolyte storage tanks and mesopores as rapid transmission channels for electrolyte ion transport. The pores with different sizes are randomly distributed and connected to each other, further promoting the diffusion rate of electrolyte ions within the pores, and improving the effective utilization of the SSA. 

With the above consideration, in this work a carbon precursor material of the LPHF was prepared by a simple method [31]. After carbonization and activated by ammonia, the LPHF possesses the ordered and opened mesoporous structure that provides an ideal matrix for obtaining hierarchical porous carbon. The thin nanobelts are intertwined with each other and show excellent conductivity. As a result, the carbon nanobelt electrodes demonstrate a large SSA (1804 to 2994 m^2^ g^−1^), high specific capacitance (maximum to 282 F g^−1^ at the current density of 0.25 A g^−1^), excellent cycle stability (the capacitance retention of 96.2% over 10,000 cycles) and rate capability.

## 2. Experiment

### 2.1. Synthesis of LPHF

Typically [31], 125 g 10 wt % hydrochloric acid, 5 g 37 wt % formaldehyde, and 1.65 g Hydroquinone were blended in a beaker for 30 min until hydroquinone was completely dissolved. The mixture was transferred into a 200 mL autoclave with Teflon lining. Then the autoclave was sealed and heated at 180 °C for 12 h in an oven. The resulting product was filtered and washed with ethanol and water. The filter production was dried at 80 °C for one night. Finally, about 4.2 g of dark brown and lightweight production was ground into powder and collected. 

### 2.2. NH_3_·H_2_O Treatment

**Figure 1:**
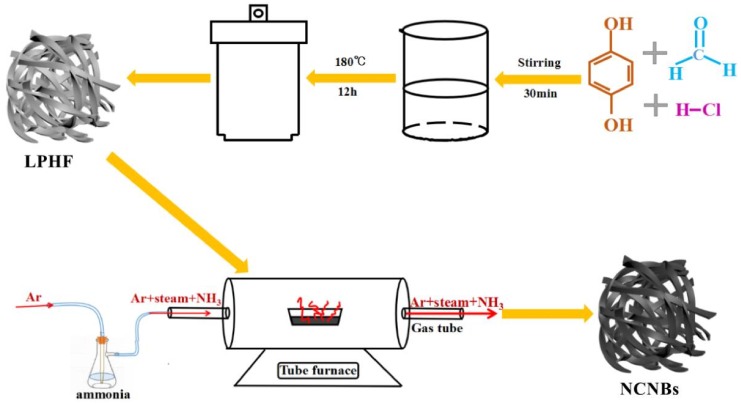
Schematic illustration of the preparation procedure of LPHF and the LPHF was treated by NH_3_·H_2_O.

The LPHF was activated and heteroatom-doped with ammonia under Ar atmosphere. The general fabrication process is illustrated in Figure 1. The ammonia solution was heated to 50 °C. The ammonia decomposed into water vapor and ammonia gas, then, the argon stream was carried by water vapor and ammonia into the tube furnace. The LPHF precursor was carbonized and activated by heating (with a ramp 5 °C/min) under a mixture of argon, ammonia, and water steam to a final temperature of 950 °C for 30 min, 45 min and 60 min. Finally, a series of nitrogen and oxygen co-doped carbon nanobelts were obtained, which were named NCNBs-30, NCNBs-45 and NCNBs-60, respectively.

### 2.3. Physicochemical Characterization

SEM images were obtained by a field emission SU-70 microscope operating at 5 kV. TEM images were recorded on a JEOL JEM2010 electron microscope (JEOL, Tokyo, Japan) operating at 200 kV. Confocal Raman spectrum was performed on an RENIDHAW invia Raman Microscope (RENIDHAW, London, UK) equipped with an Argon-ion laser (514.5 nm). N_2_ adsorption/desorption experiments were conducted at 77 K using an ASAP 2020 (Micromeritics Co. Norcross, GA, USA). The SSA was obtained according to the Brunauer-Emmett-Teller (BET) method; the total pore volume was obtained from the date of nitrogen adsorbed at P/P_0_ = 0.99. The pore volumes and pore size distribution was calculated by the BJH method. XPS analysis was conducted on an ULVAC-PHI 1800 spectrometer (ULVAC, Chigasaki, Japan).

### 2.4. Characterization of Electrochemical Performance

All electrochemical measurements were carried out using a standard three-electrode electrochemical cell equipped with 6 M KOH as the electrolyte. As-prepared electrode materials, PTFE binder, and acetylene black with a weight ratio of 8:1:1 were mixed together. The resulting slurry, containing about 2.0 mg NCNBs, was then affixed to a rectangular foam nickel. The electrode was placed in a vacuum drying oven and vacuum-dried at 110 °C for 12 h. The electrodes were tested by cyclic voltammetry (CV) and galvanostatic charge-discharge (GCD) on a CHI660D electrochemical instrument. The electrochemical impedance spectroscopy (EIS) was tested in a frequency range of 100,000 to 0.001 Hz on a VMP-300 multichannel potentiostats. The voltage range for CV test varied from −1.0 to 0 V, with different scan rates of 5–200 mV s^−1^. The specific capacitance (*C*, A g^−1^) of the supercapacitors was calculated using the following equations [32]:

(1) C=(IΔt)/(mΔV)

where *C* (F g^−1^), *I* (A), Δ*t* (s), and ΔV (V) are the specific capacitance, discharge constant current, discharge time, and discharge voltage between Δ*t* period and *m* (g) constitute the mass of the NCNBs of the individual electrode.

All the energy density (*E*, Wh kg^−1^) of the supercapacitors were obtained via the following equations [33]:

(2) $$E=\frac{CV^{2}}{2\times 4\times 3.6}$$

where *E* (Wh kg^−1^), *C* (F g^−1^), and *V* (V) are the energy density, gravimetric specific capacitance and usable voltage after the IR drop, respectively.

## 3. Results and Discussion

**Figure 2:**
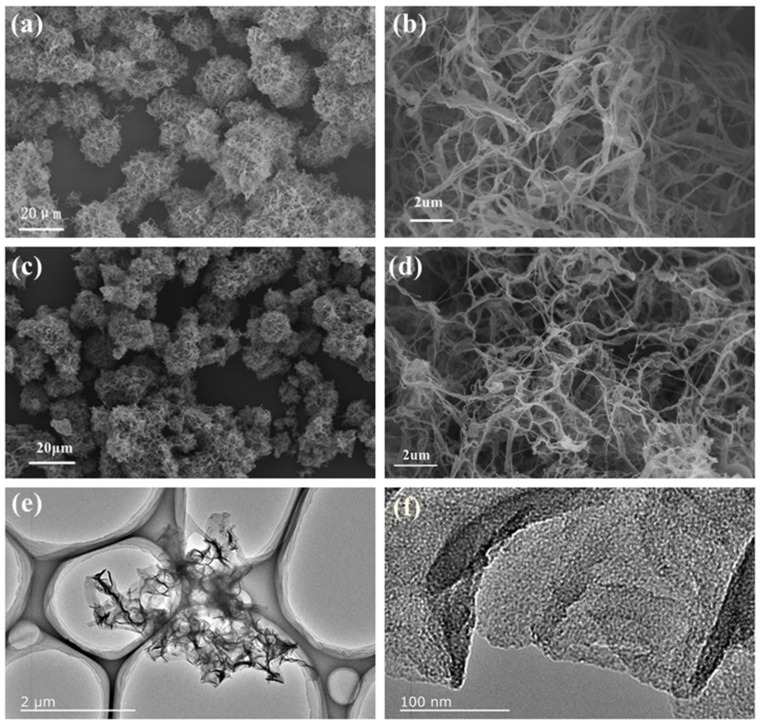
(**a**,**b**) Scanning electron microscope (SEM) images of LPHF precursor; (**c**,**d**) SEM images of NCNBs-45; (**e**,**f**) Transmission electron microscopy (TEM) images of NCNBs-45.

Figure 2 shows SEM and TEM images of LPHF and NCNBs-45. They both have obvious nanobelts random twining, and together they form a fluffy aggregate. The amplified picture of LPHF shows the width of the LPHF in the range of several tens to several hundred nanometers. The enlarged SEM images confirm that NCNBs-45 retains the original nanobelt structure of LPHF after carbonization and activation. The images of all NCNBs (Figure S1) evidently show that the nanobelts size gradually becomes smaller with the increase of carbonization time from 30 to 60 min. Figure 2e,f show TEM micrographs of NCNBs-45, a large number of mesoporous pores are distributed on the carbon nanobelts, after carbonization and activation, the nanoscale is thinner, rougher, and stretchable.

**Figure 3:**
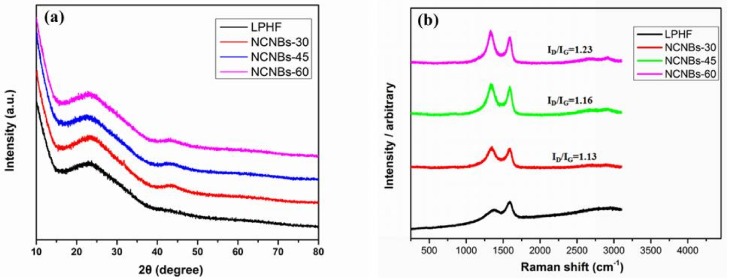
(**a**) X-ray powder diffraction (XRD) patterns of and (**b**) Raman spectraofall NCNBs.

Figure 3a shows the XRD patterns of carbonized NCNBs. Two broad diffraction peaks appeared at 2θ values of about 26° and 43°. These are the diffraction peaks of (002) and (101) planes [34] of hexagonal carbon, respectively. The two peaks are wide, indicating that these NCNBs have an amorphous feature with a low degree of graphitic crystallinity [35]. LPHF displays no diffraction peak at 43° before carbonization, after carbonization, NCNBs-30, NCNBs-45 and NCNBs-60 all possess the diffraction peaks of (101) plane, which indicates the formation of a higher degree of intra-layer condensation [35]. This is beneficial for improving the electronic conductivity of carbon materials.

It is well known that Raman spectroscopy can provide very useful information on the microstructure and crystalline ordering in carbonaceous materials. The two characteristic peaks of carbons in Raman spectra denoted as D and G bands lie at about 1350 and 1590 cm^−1^, respectively. It is generally accepted that the I_D_/I_G_ value is a reflection of defects or disorder of the carbon lattice. For amorphous carbon, I_D_/I_G_ increases as the disorder increases. As shown in Figure 3b, for the carbonized carbon nanobelts, I_D_/I_G_ value increase significantly as the carbonization time increases from 30 to 60 min, suggesting that the disorder of the NCNBs increased with the increase of activation time.

**Figure 4:**
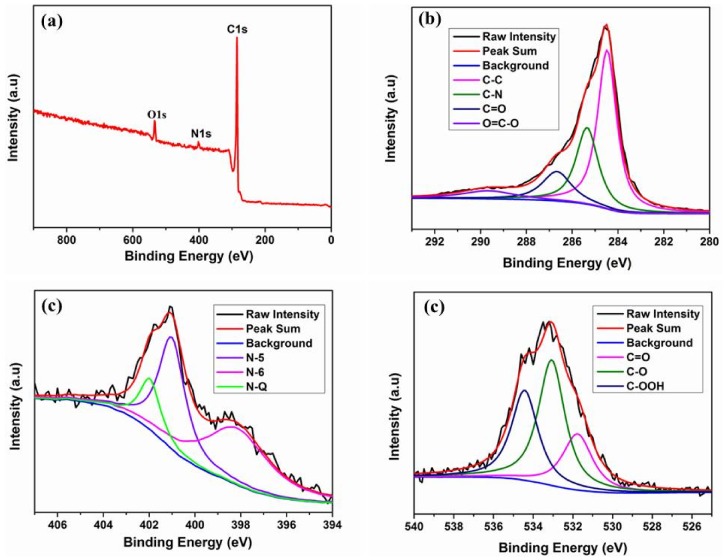
The XPS spectra of NCNBs-45 (**a**) total spectrum; (**b**) C1s XPS peaks; (**c**) N1s XPS peaks and (**d**) O1s XPS peaks.

Elemental analysis and XPS were performed on the as-prepared NCNBs. The prepared LPHF found almost no nitrogen, however, after the activation the carbon nanobelts achieved Nitrogen (Table 1). NCNBs achieved the highest nitrogen content if activated at 950 °C for 45 min. At low temperature, nitrogen was doped mainly on the surface of carbon, but at high temperature the nitrogen doping was more effective in the bulk of the carbon NCNBs-45 achieved the best nitrogen doping. XPS shows NCNBs-45 has a primary graphitic C1s peak, N1s peak and O1s peak at 285 eV, 402 eV, and 532 eV, respectively (Figure 4a). The XPS spectra show that the C1s peak of NCNBs-45 can be divided into four sub-peaks (Figure 4b), namely, C–C (284.8 eV), C–N (286.0 eV), C=O (287.2 eV), and O=C–O (289.0 eV) [6]. The N1s peak in Figure 4c can be divided into three sub-peaks at 399.1 eV, 400.0 eV, and 401.8 eV, which are ascribed to N-6 (pyridinic-N), N-5 (pyrrolic-N), and N-Q (quaternary-N), respectively [36], indicating that N was successfully doped in the NCNBs-45 after the LPHF was treated with NH_3_·H_2_O. Moreover, the three kinds of oxygen-containing functional groups were found in NCNBs-45: C=O (531 eV), C–O (533 eV), and C–OOH (534.2 eV) [37]. This indicates that the oxygen-enriched precursor and ammonia activation method used in this work can introduce abundant functional groups containing nitrogen and oxygen, which could improve the performance of the supercapacitors.

**Figure 5:**
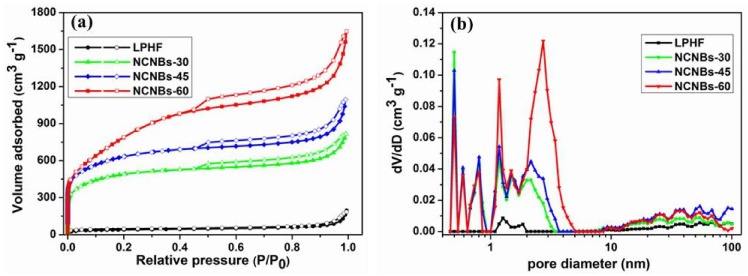
(**a**) Nitrogen adsorption-desorption isotherms and the pore size distribution curves (**b**) all of the as-prepared NCNBs.

The nitrogen adsorption-desorption isotherms are shown in Figure 5a; the isotherms of NCNBs exhibit typical characteristics of type I/IV, which indicates that the NCNBs-30, NCNBs-45, and NCNBs-60 possess tremendous micropores and mesopores [38]. Table 1 shows the surface properties and elemental analysis of all the samples. The SSA of LPHF is only 137 m^2^ g^−1^. After carbonization and activation at different times, the SSA increases to 1804 m^2^ g^−1^ for NCNBs-30, 2330 m^2^ g^−1^ for NCNBs-45, and 2994 m^2^ g^−1^ for NCNBs-60. Figure S2 shows the information about the pore volume of each sample. It is obvious that when LPHF was activated for 30 to 60 min, the micropore volume of each sample changed little (about 0.53 m^3^ g^−1^). However, it is pronounced that mesopore volume increased from 0.24 to 0.83 m^3^ g^−1^. Figure 5b showed the LPFHs possess micropores, mesopores and macropores, and as the activation time increases, the pore volume of the sample increases, implying that it is carbonization and activation of carbon nanobelts that cause the formation ofhierarchical pores, which are very beneficial for improving the capacitance and rate performance of the supercapacitors.

**Figure 6:**
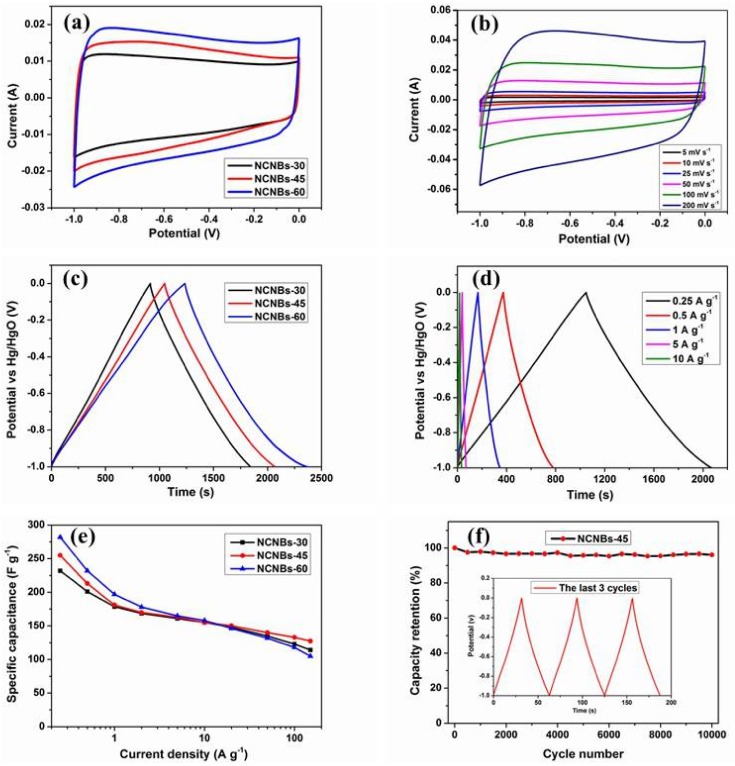
(**a**) The CV curves of NCNBs at a scan rate of 50 mV s^−1^; (**b**) CV curves of NCNBs-45at different scan rates; (**c**) GCD cures of NCNBs at 0.25 A g^−1^; (**d**) Galvanostatic charge-discharge curves at different current densities of NCNBs-45; (**e**) Specific capacitance of NCNBs electrodes at different current densities; (**f**) Cycle stability of NCNBs-45electrode at 5 A g^−1^.

Figure 6a displays the CV curves of NCNBs-30, NCNBs-45, and NCNBs-60 electrodes at a scan rate of 50 mV s^−1^ (all electrode with a loading of around 2.0 mg, the thickness of the electrodes of sample is about 50 μm). All CV curves display a quasi-rectangular shape between −1.0 and 0 V, suggesting that these carbon materials constitute typical electric double layer capacitive energy storage. As a general rule, regarding the CV profile at the same scan rate and within the same voltage window, the larger the integrated area is, the higher the specific capacitance [39], indicating that at the scan rate of 50 mV, the NCNBs-60 has the largest capacitance, and NCNBs-30 the smallest. The CV curve of NCNBs-45 electrode is shown in Figure 6b. NCNBs-45 electrode retains identical rectangle shapes at different scanning rates, even if the scanning rate was increased to 200 mV s^−1^, and NCNBs-30 and NCNBs-60 also showed the same situation (shows as Figure S3a,b) indicating that this carbon material has a stable and reproducible capacitance behavior.

GCD curves of NCNBs-30, NCNBs-45 and NCNBs-60 electrodes at a current density of 0.25 A g^−1^ (Figure 6c) show that NCNBs-60 exhibits the largest charging/discharging time, corresponding to the NCNBs-60 possessing highest capacitance at 0.25 A g^−1^. Specific capacitances calculated by Equation (1) can get 232 F g^−1^, 255 F g^−1^, and 282 F g^−1^ for NCNBs-30, NCNBs-45, and NCNBs-60 electrodes at the current density of 0.25 A g^-1^, respectively. The energy density calculated by Equation (2) can get 8.1 Wh kg^−^^1^, 8.9 Wh kg^−^^1^, and 9.8 Wh kg^−^^1^ for NCNBs-30, NCNBs-45, and NCNBs-60 electrodes at 0.25 A g^−1^. Among all the samples, NCNBs-60 shows the highest specific capacitance and energy density because of its abundant pores and the fact it possesses the largest SSA. Figure 6d shows the GCD curves of the NCNBs-45 electrode under different current densities, which remain a triangular shape even at a current density of 10 A g^−1^. NCNBs-30 and NCNBs-60 also showed the same trend (Figure S3c,d), in addition, the GCD curve of the NCNBs-45 electrode can maintain a triangular shape even at higher current densities (Figure S3e), suggesting that the activated carbon nanobelts are suitable for application in supercapacitor devices in which fast charge/discharge is needed [39]. 

Figure 6e shows the specific capacitances of NCNBs-30, NCNBs-45, and NCNBs-60 electrodes under different current densities. Though at low current densities, NCNBs-60 has the largest capacitance, when the current density exceeds 20 A g^−1^, the specific capacitance of the NCNBs-45 electrode is much larger than NCNBs-60, indicating NCNBs-45 has best rate performance. It should be noted that NCNBs-45 demonstrates a specific capacitance of 128 F g^−1^ at the current density of 150 A g^−1^ and a capability retention rate of 50.2% when the current density increases from 0.25 to 150 A g^−1^, thus, the carbon nanobelts exhibit better rate performance than most carbon materials (Table S1). Furthermore, NCNBs-45 electrode exhibits superior electrochemical stability with 96.2% capacity retention after 10,000 cycles at 5.0 A g^−1^ (Figure 6f). 

**Figure 7:**
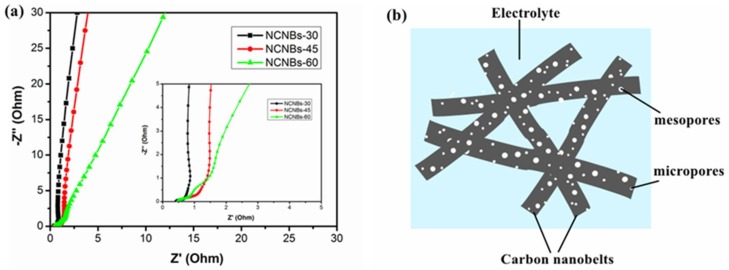
(**a**) Nyquist plots of NCNBs lectrodes and (**b**) Schematic illustration of the electrode prepared from NCNBs-45.

Figure 7a shows the Nyquist plots of the carbon nanobelt electrodes. The first intersection of the semicircle and the axis represents the equivalent series resistance (ESR) [39], which includes the resistance of the carbon materials, the electrolyte resistance, and the interface contact resistance between the active material and the collector [40]. In this study, there is no obvious difference between the ESR values of NCNBs-30 (0.43 Ω), NCNBs-45 (0.46 Ω) and LPHF-60 (0.47 Ω). While in the low frequency region, the straight lines of NCNBs-30 and NCNBs-45 are more parallel than NCNBs-60 indicating the ideal capacitive behavior of NCNBs-30 and NCNBs-45.Therefore, the superior capacitive behavior of NCNBs-45 electrode can be attributed to the high nitrogen content, high SSA and the synergetic effect of the hierarchically porous structure of carbon material. 

The schematic illustration of the NCNBs electrode is shown in Figure 7b; the excellent electrochemical performance of NCNBs was attributed principally to the following features [41]: (1) The highly conductive carbon nanobelts tangle up in a random manner to form a three-dimensional conductive network; (2) The synergetic effect of hierarchical pores is the key reason for the high capacitance and the excellent rate capability [42,43]; (3) The short ion diffusion pathway exists owing to the thinness of the carbon nanobelts (~20 nm); (4) NCNBs are doped with appropriate amounts of nitrogen and oxygen, which increases the wettability of the carbon surface towards the aqueous electrolyte and consequently improves the capacitive performances [44,45].

## 4. Conclusions

In summary, a simple method was used to prepare carbon nanobelts that were activated with aqueous ammonia at different times, and the resultant carbon nanobelts possess high SSA, abundant micropores and mesopores. The synergistic effect of the hierarchical pores structure contributes to the transmission and diffusion of ions, and the appropriate nitrogen and oxygen co-doped improves the wettability and electrical conductivity of the carbon materials, which are beneficial for improving the performance of the supercapacitors. Due to NCNBs-60 having the highest SSA (2994 m^2^ g^−1^), it shows a high specific capacitance of 282 F g^−1^ at 0.25 A g^−1^ in 6 M KOH electrolyte solution. Although the capacitance of the NCNBs-45 electrode is slightly less than NCNBs-60 at low current density, it has a remarkable rate performance, and the electrode maintains 128 F g^−1^ at a high current density of 150 A g^−1^. In addition, NCNBs-45 also exhibits an excellent electrochemical stability (less than 4% degradation after 10,000 cycles at 5 A g^−1^). This work demonstrates that carbon nanobelt can be a potential electrode material of supercapacitors with high-performance in energy storage.

## Figures and Tables

**Table 1:** Surface properties and element analysis of NCNBs.

Sample	S_BET_ (m^2^ g^−1^)	S_DFT_ (m^2^ g^−1^)	Pore Volume Fraction (cm^3^ g^−1^)			Element Analysis		
			V_total_	V_mic_	V_mes_	C (wt %)	N (wt %)	O (wt %)
LPHF	137	82	0.19	0.03	0.06	66.49	--	24.36
NCNBs-30	1804	1421	0.83	0.52	0.24	93.34	3.02	7.93
NCNBs-45	2330	1620	1.10	0.53	0.40	92.50	2.65	6.64
NCNBs-60	2994	1875	1.47	0.54	0.83	92.61	2.36	5.57

## Supplementary Materials

The following are available online at http://www.mdpi.com/1996-1944/11/4/556/s1, Figure S1: The SEM image of the (a) LPHF, (b) NCNBs-30, (c) NCNBs-45 and (d) NCNBs-60, Figure S2: Micropore and mesopore volume of all samples, Figure S3: (a) The CV curves of NCNBs-30 and (b) NCNBs-60 at different scan rates, (c) Galvanostatic charge-discharge curves at different current densities of NCNBs-30 and (d) NCNBs-60, (e) Galvanostatic charge-discharge curves of NCNBs-45 at high current densities, Table S1: Comparison of specific capacitance with various porous carbon electrode.
