# Supplementary material for: Facile Synthesis of Nitrogen and Oxygen Co-Doped Clews of Carbon Nanobelts for Supercapacitors with Excellent Rate Performance
Source: Materials (Basel). 2018 Apr 4;11(4):556. doi: 10.3390/ma11040556 (PMC5951440; doi:10.3390/ma11040556)
Supplement: Supplementary file 1 [file materials-11-00556-s001.pdf]

Supplementary Materials: Facile Synthesis of Nitrogen and Oxygen Co-Doped Clews of Carbon Nanobelts for Supercapacitors with Excellent Rate Performance

Liang Yu, Shaozhong Zeng, Xierong Zeng, Xiaohua Li, Hongliang Wu, Yuechao Yao, Wenxuan Tu, and Jizhao Zou

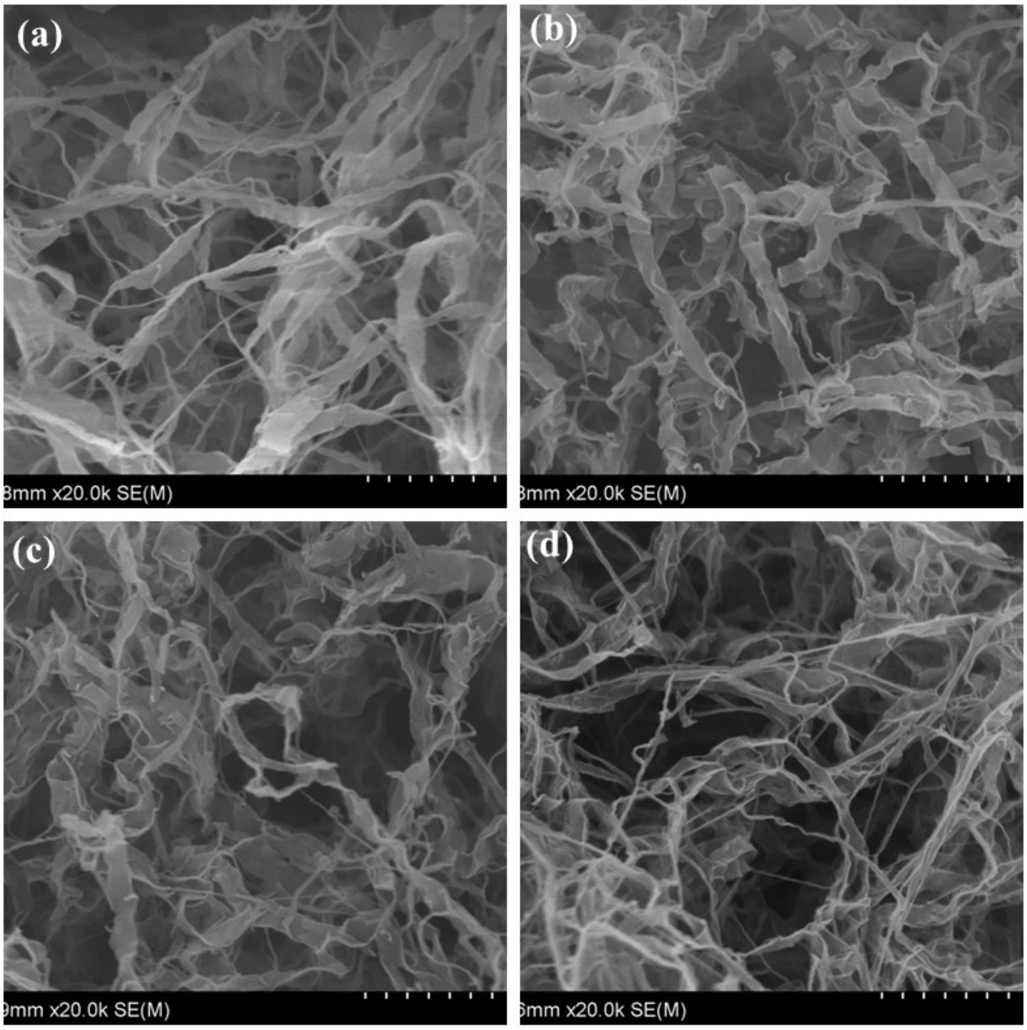

Figure S1. The SEM image of the (a) LPHF, (b)NCNBs-30, (c) NCNBs -45 and (d) NCNBs -60.

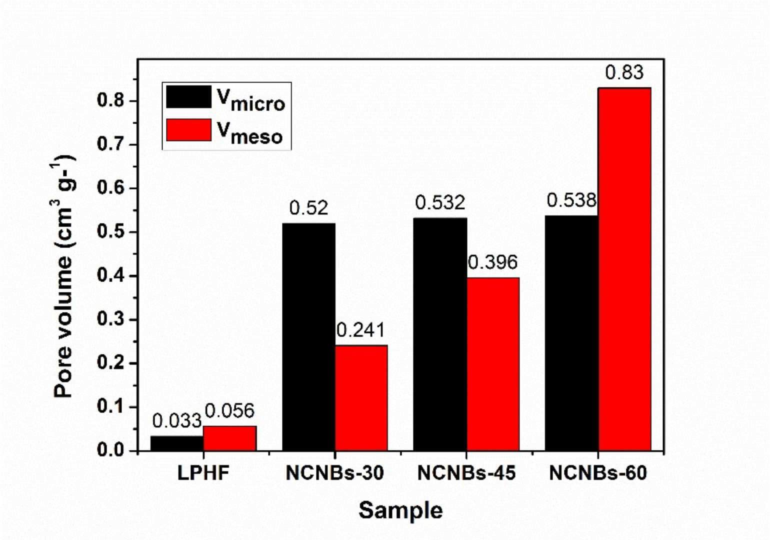

Figure S2. Micropore and mesopore volume of all samples.

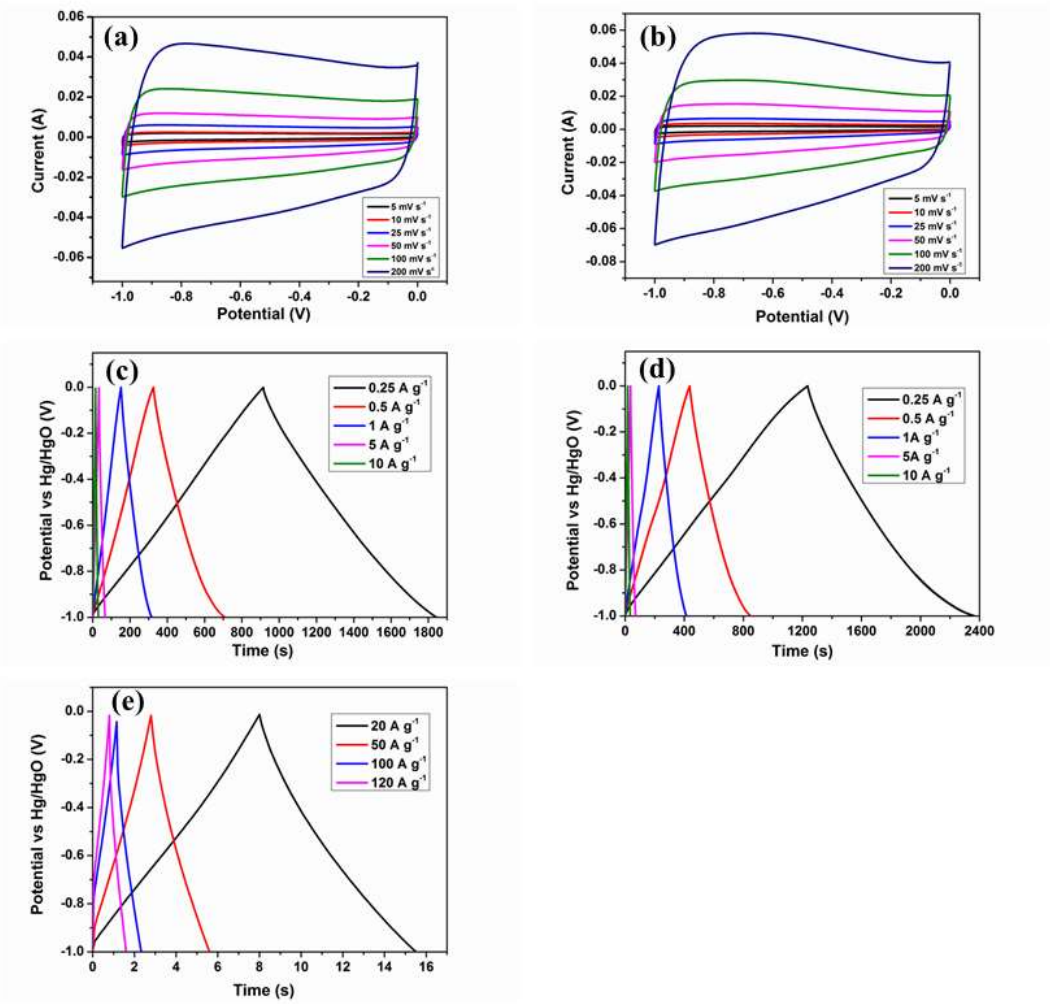

Figure S3. (a) The CV curves of NCNBs-30 and (b) NCNBs-60 at different scan rates, (c) Galvanostatic charge-discharge curves at different current densities of NCNBs-30 and (d) NCNBs-60, (e) Galvanostatic charge-discharge curves of NCNBs-45 at high current densities.

Table S1. Comparison of specific capacitance with various porous carbon electrode.

Electrode Materials	Electrolyte	Specific Capacitance (F g^{-1})	Ref.
N-doped HPC	6M KOH	260 F g^{-1}/0.2 A g^{-1}, 114 F g^{-1}/40 A g^{-1}	[1]
KOH-activated nitrogen doped porous carbon nanowires	1M H2SO	291/1 A g^{-1}, 150 F g^{-1}/10 A g^{-1}	[2]
Graphene-based nitrogen self-doped HPC aerogels	6M KOH	197 F g^{-1}/0.2 A g^{-1}, 108 F g^{-1}/10 A g^{-1}	[3]
Activated carbon nano-onion prepared using 6M KOH	2M KNO_3	126.3 F g^{-1}/0.75 A g^{-1},89.6 F g^{-1}/25 A g^{-1}	[4]
Graphene-beaded carbon nanofibers	6M KOH	263.7 F g^{-1}/0.1 A g^{-1},131.3 F g^{-1}/2.5 A g^{-1}	[5]
N-doped microporous carbon/carbon nanotubes	1M H_2SO_4	162 F g^{-1}/0.5 A g^{-1},140 F g^{-1}/2A g^{-1}	[6]
Hierarchical porous carbon aerogel derived from bagasse	6M KOH	142 F g^{-1}/0.5 A g^{-1},90 F g^{-1}/10A g^{-1}	[7]
3D hexaporous Carbon	1M H_2SO_4	154 F g^{-1}/0.5 A g^{-1},123 F g^{-1}/10A g^{-1}	[8]
Microporous Carbon Nanoplates	1M H_2SO_4	264 F g^{-1}/0.1 A g^{-1}, 105 F g^{-1}/70A g^{-1}	[9]
Three-dimensional hierarchical porous carbon	6M KOH	236.3 F g^{-1}/0.2 A g^{-1}, 152.5 F g^{-1}/30A g^{-1}	[10]

References

[1] J. Zhou, M. Wang, H. Wang, Q. Chi, Comparison of two nutrition assessment tools in surgical elderly inpatients in Northern China, Nutr. J. 14 (2015) 68. doi:10.1186/s12937-015-0054-8.

[2] B. Wang, J. Qiu, H. Feng, E. Sakai, T. Komiyama, KOH-activated nitrogen doped porous carbon nanowires with superior performance in supercapacitors, Electrochim. Acta. 190 (2016) 229–239. doi:10.1016/j.electacta.2016.01.038.

[3] P. Hao, Z. Zhao, Y. Leng, J. Tian, Y. Sang, R.I. Boughton, C.P. Wong, H. Liu, B. Yang, Graphene-based nitrogen self-doped hierarchical porous carbon aerogels derived from chitosan for high performance supercapacitors, Nano Energy. 15 (2015) 9–23. doi:10.1016/j.nanoen.2015.02.035.

[4] Y. Gao, Y.S. Zhou, M. Qian, X.N. He, J. Redepenning, P. Goodman, H.M. Li, L. Jiang, Y.F. Lu, Chemical activation of carbon nano-onions for high-rate supercapacitor electrodes, Carbon N. Y. 51 (2013) 52–58. doi:10.1016/j.carbon.2012.08.009.

[5] Z. Zhou, X.F. Wu, Graphene-beaded carbon nanofibers for use in supercapacitor electrodes: Synthesis and electrochemical characterization, J. Power Sources. 222 (2013) 410–416. doi:10.1016/jjpowsour.2012.09.004.

[6] K.S. Kim, S.J. Park, Synthesis and high electrochemical capacitance of N-doped microporous carbon/carbon nanotubes for supercapacitor, J. Electroanal. Chem. 673 (2012) 58–64. doi:10.1016/j.jelechem.2012.03.011.

[7] P. Hao, Z. Zhao, J. Tian, H. Li, Y. Sang, G. Yu, H. Cai, H. Liu, C.P. Wong, A. Umar, Hierarchical porous carbon aerogel derived from bagasse for high performance supercapacitor electrode, Nanoscale. 6 (2014) 12120–12129. doi:10.1039/C4NR03574G.

[8] P. Yadav, A. Banerjee, S. Unni, J. Jog, S. Kurungot, S. Ogale, A 3D hexaporous carbon assembled

from single-layer graphene as high performance supercapacitor, ChemSusChem. 5 (2012) 2159–2164. doi:10.1002/cssc.201200421.

[9] Y.S. Yun, S.Y. Cho, J. Shim, B.H. Kim, S.J. Chang, S.J. Baek, Y.S. Huh, Y. Tak, Y.W. Park, S. Park, H.J. Jin, Microporous carbon nanoplates from regenerated silk proteins for supercapacitors, Adv. Mater. 25 (2013) 1993–1998. doi:10.1002/adma.201204692.

[10] L. Qie, W. Chen, H. Xu, X. Xiong, Y. Jiang, F. Zou, X. Hu, Y. Xin, Z. Zhang, Y. Huang, Synthesis of functionalized 3D hierarchical porous carbon for high-performance supercapacitors, Energy Environ. Sci. 6 (2013) 2497. doi:10.1039/c3ee41638k.

© 2018 by the authors. Licensee MDPI, Basel, Switzerland. This article is an open access article distributed under the terms and conditions of the Creative Commons Attribution (CC BY) license (http://creativecommons.org/licenses/by/4.0/).
